# Development of simple, scalable protease production from *Botrytis cinerea*

**DOI:** 10.1007/s00253-022-11817-1

**Published:** 2022-02-16

**Authors:** Rachel A. Self, Mark D. Harrison, Valentino S. Te’o, Steve Van Sluyter

**Affiliations:** 1 Centre for Agriculture and the Bioeconomy, Queensland University of Technology, Brisbane, QLD 4000 Australia; 2 School of Biology and Environmental Science, Queensland University of Technology, Brisbane, QLD 4000 Australia; 3 Department of Biological Sciences, Macquarie University, Sydney, NSW 2109 Australia

**Keywords:** Haze-forming proteins, Laccase, Glucan, Fermentation, Enzymes

## Abstract

**Abstract:**

Heat haze-forming proteins are stable during winemaking and are typically removed via adsorption to bentonite. Proteolytic degradation is an alternative method to prevent wine-haze and offers the opportunity to reduce the environmental impacts and labor cost of the process. Herein, we describe the development of a production system for *Botrytis cinerea* proteases for the enzymatic degradation of heat haze-forming proteins. The effect of culture medium on the secretion of glucan by *B. cinerea* was investigated and methods to inactivate *B. cinerea* laccase in liquid culture medium were assessed. Protease production by *B. cinerea* was scaled up from 50 mL in shake flasks to 1 L in bioreactors, resulting in an increase in protease yield from 0.30 to 3.04 g L^−1^. Glucan secretion by *B. cinerea* was minimal in culture medium containing lactose as a carbon source and either lactic or sulfuric acid for pH control. *B. cinerea* laccases were inactivated by reducing the pH of culture supernatant to 1.5 for 1 h. *B. cinerea* proteases were concentrated and partially purified using ammonium sulfate precipitation. SWATH-MS identified aspartic acid protease BcAP8 amongst the precipitated proteins. These results demonstrate a simple, affordable, and scalable process to produce proteases from *B. cinerea* as a replacement for bentonite in winemaking.

**Key points:**

• *Isolates of B. cinerea that produce proteases with potential for reducing wine heat-haze forming proteins were identified.*

• *Media and fermentation optimization increased protease yield tenfold and reduced glucan secretion.*

• *Low pH treatment inactivated laccases but not proteases.*

**Graphical abstract:**

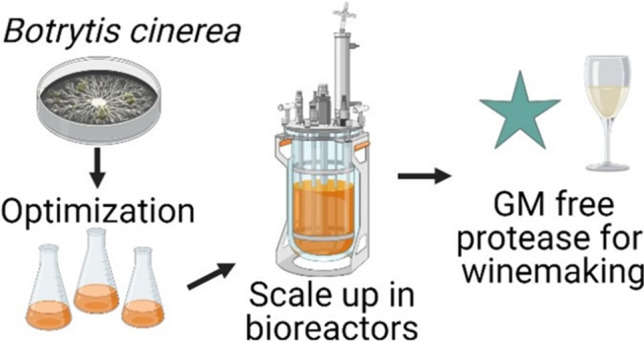

**Supplementary Information:**

The online version contains supplementary material available at 10.1007/s00253-022-11817-1.

## Introduction

Bentonite is a clay that is added to wine after fermentation to adsorb heat-haze forming proteins, including chitinases and thaumatin-like proteins (Waters et al. 1996). These proteins come from grapes and aggregate when heated, causing an undesirable haze in the wine (Marangon et al. 2009). While bentonite is commonly used in winemaking (Logan 2018), it has many disadvantages, including the loss of up to 10% of wine volume without the addition of a filtering step for recovery (Majewski et al. 2011), relatively high costs of labor during treatment and disposal of bentonite waste (Waters et al. 2005), and the loss of finer sensory characteristics from wines (Vela et al. 2017). As a result, there is a need to develop an alternative to bentonite for the removal of heat-haze forming proteins during winemaking.

Alternative techniques for removal of heat-haze forming proteins from wine have been identified, including ultrafiltration (Flores et al. 1990), non-bentonite absorbents (Sarmento et al. 2000) including zirconium oxide (Marangon et al. 2011) and carrageenan (Ratnayake et al. 2019), but none has been adopted by commercial winemakers. The combination of a mixture of Aspergillopepsin I and II (proteases) and flash pasteurization successfully heat-stabilized white wine without adverse effects on sensory characteristics (Marangon et al. 2012) and at lower operating cost relative to the addition of bentonite (Logan 2015); however, the equipment needed for the additional processing step (flash pasteurization) required for enzyme activation was relatively expensive. Therefore, the ideal protease for removal of heat-haze forming proteins in white wine would be active at normal winemaking temperatures and would not require additional heating or activation. Microbial proteases are used in a wide range of industrial processes and have a wide range of physicochemical characteristics, including optimal temperature (Mienda et al. 2014).

*B. cinerea* is one of the most well-studied fungi (Van Kan 2006) and infects more than 200 crop species (Miclea and Puia 2012), including grape vines. *B. cinerea* secretes a complex mixture of proteins (Espino et al. 2010; Fernández-Acero et al. 2007; González-Fernández et al. 2014; Shah et al. 2009), including metalloproteases and serine proteases, as well as a wide range of enzymes involved in processes such as cell-wall degradation, oxidative burst, and toxin production (González-Fernández et al. 2015). The most abundant proteins in the *B. cinerea* secretome are a family of aspartic acid proteases designated BcAP1–BcAP14 (Ten Have et al. 2004, 2010). BcAP8 alone can represent up to 23% of the total protein secreted by *B. cinerea* (Ten Have et al. 2010) and recombinant BcAP8 expressed in transgenic *Pichia pastoris* has been used to reduce heat-haze in white wine (Van Sluyter et al. 2013). While the use of recombinant BcAP8 to reduce wine heat-haze has been demonstrated, genetically modified products are not accepted in winemaking (Chambers and Pretorius 2010) and a process to produce native *B. cinerea* protease is required.

Relatively large amounts of proteases are secreted when *B. cinerea* is cultured in liquid medium (González-Fernández et al. 2015), in addition to laccases (Van Kan et al. 2016) and β-(1,3)(1,6)-D-glucans (Bar-Nun et al. 2007). Laccases are multicopper oxidases that significantly reduce wine quality (Steel et al. 2013; Vignault et al. 2020) and are relatively stable under winemaking conditions. Inactivation of laccases during winemaking has only been observed with the addition of high concentrations of sulfite (> 200 mg L^−1^) or heat-treatment of wine (Claus 2017). Several low-molecular weight compounds have shown to reduce or eliminate laccase production by *B. cinerea* in culture, specifically ethylenediaminetetraacetic acid (EDTA) and calcium chloride (CaCl_2_). The addition of 12 mM EDTA to *B. cinerea* culture medium significantly reduced laccase formation (Bar-Nun et al. 1988) while the addition of 13–20 mM EDTA partially inhibited laccase activity (Sansone et al. 2011; Zouari et al. 1987). The addition of CaCl_2_ to culture medium significantly decreased detected laccase levels, at relatively low concentrations (80 and 186 mg L^−1^ respectively). It has further been shown that concentrations of CaCl_2_ up to 16 g L^−1^ have little negative effect on mycelial growth or total protein secretion in *B. cinerea* (Chardonnet et al. 2000).

The extracellular β-(1,3)(1,6)-D-glucan “sheath” that *B. cinerea* secretes aids and protects the fungus during host colonization (Gil-ad et al. 2001) and also encapsulates a sub-set of secreted *B. cinerea* proteins and lipids (Doss 1999). The presence of this glucan sheath increases viscosity of *B. cinerea* culture media and interferes with subsequent analysis of media chemistry and composition (Pielken et al. 1990). The choice of carbon source used in *B. cinerea* culture medium influences glucan secretion (Leal et al. 1979). Monosaccharides are commonly used as a carbon sources for *B. cinerea* fermentation (Ciliberti et al. 2016; Cotoras et al. 2009) and, in such fermentation systems, glucan secretion up to 13 g L^−1^ has been observed (Pielken et al. 1990). However, when lactose (a disaccharide) was used as a carbon source, glucan production was not observed (King et al. 1969).

In the present work, we screened eighty-six wild-type *B. cinerea* isolates for their capacity to secrete protease and laccase on solid growth medium. Three strains were down selected and evaluated for their capacity to secrete protease in liquid medium in shake flasks. The effect of media composition on glucan production was evaluated and media treatments to reduce laccase activity were investigated. Production of the elite *B. cinerea* strain was scaled up to 1 L and a process was developed to produce a crude protease preparation without laccase activity. The results described herein suggest that *B. cinerea* has potential as a source of protease for removal of heat-haze forming proteins in wine, thereby providing a practical alternative to bentonite.

## Materials and methods

### *Botrytis cinerea* isolates

Wild-type isolates of *B. cinerea* (*n* = 16) collected from grape vines in Victoria, South Australia, and New South Wales, Australia, were provided by Treasury Wine Estates (Nuriootpa, South Australia). Isolates (*n* = 5) previously collected from SA and Fernhill, VIC, were purchased from AgPath (Vervale, Victoria), and 65 isolates were provided by the Primary Industries and Regions South Australian division of the SA Research and Development Institute (PIRSA-SARDI; Adelaide, South Australia), collected from the Adelaide Hills, SA. *B. cinerea* RV05 (accession #BRIP 74,436 a), AH42 (accession #BRIP 74,435 a), and AH55 (accession #BRIP 74,434 a) are available from Queensland Plant Pathology Herbarium, Department of Agriculture and Fisheries, Brisbane, Australia.

The *B. cinerea* reference strain (B05.10; Available from ICMP, New Zealand, Strain no. ICMP 14,168), a haploid wild-type strain originally isolated from grapes (Büttner et al. 1994) and now used in laboratories worldwide (Amselem et al. 2011; Liñeiro et al. 2018; Lovato et al. 2019; Quijada‐Morin et al. 2018; Srivastava et al. 2020; Zhou et al. 2018), was provided by Dr Jan Van Kan from Wageningen Agricultural University, Wageningen, Netherlands.

### Cultivation medium and chemicals

*B. cinerea* isolates were cultivated on potato dextrose agar (Oxoid, Hampshire, UK) and sub-cultured from hyphal plugs taken from agar plates or from spore stocks (12.4% (v/v) glycerol and 0.04% (v/v) Tween 80). Soybean flour type I, α-lactose monohydrate, DL-malic acid, L-( +)-lactic acid solution ≥ 85%, EDTA, and CaCl_2_ were purchased from Sigma-Aldrich, St Louis, USA. Sulfuric acid 98% and NaOH were purchased from Chem-supply (Gillman, Australia). Food grade ammonium sulfate was purchased from Merck (Darmstadt, Germany) and tartaric acid (food grade) was purchased from McKenzie’s, Altona, Australia. All reagents were of analytical grade unless indicated. All solutions were prepared using ultrapure water (Milli-Q system, Merck, Darmstadt, Germany).

Standard soybean flour (SBF) medium contained 2% (w/v) soybean flour and 1% (w/v) lactose in minimal salts base (1 g L^−1^ KH_2_PO_4_, 0.5 g L^−1^ K_2_HPO_4_, 0.5 g L^−1^ MgSO_4_‧7H_2_O, 0.5 g L^−1^ KCl, and 0.01 g L^−1^ FeSO_4_). The pH of media was adjusted by the addition of malic or lactic acid (Merck, Darmstadt, Germany).

### Screening protease and laccase production from *B. cinerea* on agar plates

*B. cinerea* isolates were cultured on skim milk agar (Rajamani and Hilda 1987) at pH 3.5 to identify those isolates that secreted proteases active at the acidic pH of wine. Skim milk agar contained 2% (w/v) skim milk powder (Coles, Glen Iris, Australia), 0.5% (w/v) yeast extract, and 1.5% (w/v) agar, with 0.1% Triton X-100 (v/v) to limit the spread of fungal hyphae during growth (Nevalainen et al. 2014). Additives were prepared in a minimal salt solution (1 g KH_2_PO_4_, 0.5 g K_2_HPO_4_, 0.5 g MgSO_4_‧7H_2_O, 0.5 g KCl, and 0.01 g FeSO_4_ per liter in dH_2_O) (Cotoras et al. 2009). Laccase secretion from isolates was analyzed on guaiacol agar plates, containing potato dextrose agar with 0.02% (v/v) guaiacol (Sigma-Aldrich, St Louis, USA) (Kiiskinen et al. 2004).

All plates were inoculated by pushing a 1-µL loop of mycelium from a 7- to 14-day-old potato dextrose agar culture of *B. cinerea* into the surface of the agar and incubated at 22 °C. Proteolysis was evident on skim milk agar by a zone of clearing in the opaque agar under, and around the edge of, a growing colony. Laccase secretion was indicated on guaiacol agar plates by a developing color change from faint red to deep brown. Table 1 outlines the scoring metric used to analyze and compare protease and laccase secretion by the *B. cinerea* isolates.

**Table 1:** Scale used for scoring zone of clearing on skim milk agar and area of color change on guaiacol agar for analysis of protease and laccase production from *B. cinerea* isolates

Skim milk agar		Guaiacol agar	
Score	Description	Score	Description
0	No visible zone of clearing	0	No visible coloring in agar
1	Clearing just under colony	1	Slight coloring, typically just under colony
2	Clearing up to 2 mm from colony	2	Stronger and more even coloring to the agar
3	Clearing > 2 mm from colony	3	Dramatic, dark coloring to the agar

### Shake flask fermentation

Shake flask cultivation was undertaken by inoculating 1 × 10^5^ *B. cinerea* spores in 250-mL Erlenmeyer flasks in the dark using 50-mL culture volumes, agitation at 128 rpm, and a temperature of 22 °C. All experiments were performed with three biological replicates. Samples of supernatant were collected immediately after inoculation and after 1, 3, 5, and 7 days of cultivation. Samples of supernatant were immediately snap frozen in liquid N_2_ and stored at − 80 °C until analyzed.

Comparison of protease secretion between wild-type *B. cinerea* isolates was performed in SBF medium. Inhibition of laccase secretion by *B. cinerea* in liquid cultures was analyzed with added EDTA and CaCl_2_. Isolates were cultured in SBF with EDTA added at 20, 60, or 80 mM, and CaCl_2_ was added at 1.7, 72.1, or 144.6 mM (0.186, 8, and 16 g L^−1^). The impact of addition of organic acids (for pH control) on glucan secretion by *B. cinerea* was evaluated using SBF medium containing 50 mM malic acid, or either 50 or 100 mM lactic acid.

### Protease activity assay

Protease activity in *B. cinerea* culture medium was quantified using the EnzChek® Protease Assay Kit, green fluorescence, targeting metallo-, serine-, acid-, and sulfhydryl proteases (Thermo-Fisher, Waltham, MA, USA). Briefly, aliquots of fermentation supernatant were diluted (between 1/200 and 1/2000) in 100 mM lactate buffer, pH 3.5. Standards were prepared using pepsin from porcine gastric mucosa (Sigma-Aldrich, St Louis, MO, USA) containing 0.39, 0.78, 1.56, 3.13, 6.25, 12.5, 25, and 50 mg L^−1^. Samples, standards, and controls (50 µL) were analyzed in triplicate on black, flat bottomed 96-well plates (Greiner CELLSTAR®, Sigma-Aldrich, St Louis, USA). The EnzChek® substrate was kept at ~ 4 °C before addition to the microplate and added immediately before analysis (50-µL volume).

Fluorescence of the reaction was measured every min for 10 min at 485-nm excitation and 530-nm emission on a FLUOstar OPTIMA microplate reader (BMG Labtech, Ortenberg, Germany). A second-order polynomial standard curve was calculated from the fluorescent kinetics of pepsin standards, and protease activity in the *B. cinerea* culture medium was calculated as pepsin equivalents (corrected for dilution factor). The protease activity was averaged across three biological replicates, and the standard deviation calculated using Microsoft Excel software (Redmond, USA).

### Laccase activity assays

Laccase activity in *B. cinerea* medium was measured using an adaptation of the method described by Li et al. (2008) using guaiacol as a substrate. Briefly, a 2 mM solution of guaiacol was prepared in 10 mM acetate buffer, pH 3.5. Sub-samples of culture supernatant were diluted with 10 mM acetate buffer, pH 3.5. Sub-samples of diluted culture supernatant (33 µL) were mixed thoroughly with 2 mM guaiacol solution (167 µL) in 96-well microplates. The mixtures were incubated at room temperature (~ 22 °C) for 2 h and the absorbance at 544 nm was measured using a FLUOstar OPTIMA microplate reader (BMG Labtech, Ortenberg, Germany). The absorbance at 544 nm in microplate wells containing aliquots (33 µL) of 10 mM acetate buffer, pH 3.5, was averaged and subtracted from the absorbance measured in microplate wells containing sub-samples of diluted culture supernatant. The laccase activity in aliquots (33 µL) of *Rhus vernicifera* laccase (Sigma-Aldrich, St Louis, USA) solutions of known concentration was measured and the results used to determine the laccase activity in diluted culture supernatant. Aliquots of sterile culture supernatant (33 µL) and grape juice were analyzed as negative controls in each laccase activity assay. All samples were analyzed in triplicate and laccase activity was presented as a mean with a standard deviation.

Re-activation of *B. cinerea* laccases was evaluated using the 2 mM guaiacol solution described above. Briefly, sub-samples of *B. cinerea* fermentation media and crude protease preparations that had been treated to deactivate laccase were diluted 1/20 using Chardonnay grape juice. Aliquots (33 µL) of diluted fermentation media and crude protease preparations were mixed with 2 mM guaiacol solution (167 µL) in 96-well microplates, and the absorbance at 544 nm was measured. The microplates were incubated at 24 °C overnight (~ 18 h) and the absorbance at 544 nm in each microplate well was measured. The change in absorbance over 24 h was used to identify samples in which laccase activity was present. Results were blank corrected against pure Chardonnay grape juice before calculating the average and standard deviation.

### Bioreactor fermentation

New Brunswick TM Bioflo®/CelliGen® 115 Fermenter and Bioreactor systems (Eppendorf 2012) were used to scale protease production to 1 L. Fermenters were operated in batch mode in heat-blanketed borosilicate vessels, with a working volume of 800 mL in 1 L bioreactors. Fermentation media consisted of 2% (w/v) soybean flour, 1% (w/v) lactose, and 100 mM lactic acid in minimal salts medium and were sterilized at 121 °C for 60 min. Lactose solution (1 g L^−1^) was sterilized at 121 °C for 15 min separately to the fermentation media to minimize caramelization. Foaming during fermentation was controlled by the addition of Struktol® J 673 A anti-foam (Schill + Seilacher “Struktol” GmbH, Hamburg, Germany) as required, and fermentation medium pH was maintained by the addition of 2 N lactic or 2 N sulfuric acid, and 3 N KOH or 3 N NaOH. One-way ANOVA statistical analyses were performed using Microsoft Excel software (Redmond, USA).

### Bioreactor operating conditions

Bioreactors were operated under the following baseline conditions: agitation 300 rpm, pH (maximum) 4.0, aeration at 1.0 VVM (0.8 SLPM), dO_2_ (minimum) 20%, and temperature 24 °C. A cascade was set to control dO_2_ by increasing agitation to a maximum of 500 rpm and aeration rate to maximum of 2 VVM (vessel volume per minute) (1.6 SLPM (standard liters per minute)).

### Laccase inactivation

The inactivation of laccase at low pH was analyzed by drop-wise addition of ice-cold sulfuric acid (1 M; Chem-supply, Seventeen Mile Rocks, Australia) to portions of *B. cinerea* culture supernatant. Addition was performed on ice to prevent heat denaturation of proteases, and pH was constantly monitored while the supernatant was stirred. A volume of water equivalent to the volume of added acid was added to control samples to account for sample dilution. Low pH samples were kept at 4 °C for 1 h to allow time for inactivation of laccase. Samples were placed back onto ice and the pH returned to 4 with the addition of ice-cold 1 M NaOH (Chem-supply, Seventeen Mile Rocks, Australia). All analyses were performed in triplicate.

### SDS-PAGE analysis

Proteins were precipitated from supernatant samples using the method described by Wessel and Flügge (1984), and reconstituted in 40 µL master mix containing (v/v) 70% dH_2_O, 25% 4 × NuPAGE™ lithium dodecyl sulfate sample buffer (Thermo-Fisher, Waltham, MA, USA), and 5% *β*-mercaptoethanol (≥ 99%; Merck, Darmstadt, Germany). Samples were heated at 90 °C for 5 min and centrifuged at 8000 rpm for 5 min at 8 °C to pellet insoluble contents. Samples were loaded onto 15-well Bolt® 4 — 12% Bis–Tris plus gels (Thermo-Fisher, Waltham, MA, USA) and resolved by electrophoresis at 90 V for 40 min. SeeBlue™ Plus2 Pre-stained Protein Standard (Thermo-Fisher, Waltham, MA, USA) or Precision Plus Protein Dual Color Standard (Bio-Rad, Hercules, CA, USA) (5 µL) was resolved on each gel to enable estimation of protein molecular mass. Gels were electrophoresed in 1X Bolt® MES SDS Running Buffer (Thermo-Fisher, Waltham, MA, USA) in a Mini Gel Tank (Thermo-Fisher, Waltham, MA, USA) with 10 mg sodium metabisulfite added to gel tank reservoir to improve definition of protein bands. Gels were fixed with 10% (v/v) acetone, 40% (v/v) ethanol with agitation for 15 min, then stained overnight in QC Colloidal Coomassie Stain (Bio-Rad, Hercules, CA, USA) and de-stained for 3 h with at least 3 changes of dH_2_O.

Stained gels were placed onto a light box for photographing with a Samsung (Suwon, Korea) mobile phone camera. Pictures of gels were cropped, changed to black and white, sharpened (+ 10 contrast, + 10 brightness), and annotated using paint.net software (available for free download at https://www.getpaint.net/).

### Analyzing ammonium sulfate precipitation of *B. cinerea* secreted proteins

Precipitation of *B. cinerea* proteases was analyzed at 0, 40, 60, and 80% (w/v) saturation of ammonium sulfate, in triplicate. Ammonium sulfate (Merck, Darmstadt, Germany) was ground into a fine powder with a mortar and pestle before use to increase the rate of dissolution (Burgess 2009). Ammonium sulfate was incrementally added to 10-mL volumes of protease-containing supernatant (with laccase inactivated by pH reduction). Samples were kept on ice for the duration of ammonium sulfate addition to prevent protease denaturation, and supernatants were stirred gently (~ 200 rpm) until the ammonium sulfate was fully dissolved. Proteases were precipitated from solution overnight at 4 °C and then collected by centrifugation at 10,000 rpm for 30 min at 0 °C. Precipitated proteases were dissolved in 10 mM tartrate buffer, pH 3.5, and stored at − 80 °C. Larger scale precipitation was analyzed with 100-mL volumes at 80% ammonium sulfate saturation, using the method described above.

### Protein mass spectrometry and data analysis

The secreted *B. cinerea* proteins precipitated by ammonium sulfate and re-dissolved in tartrate buffer were identified by Sequential Window Acquisition of All Theoretical Mass Spectra (SWATH-MS) data-independent acquisition as per the method described by Chemonges et al. (2017). Briefly, five volumes of acetone were added to *B. cinerea* protein solutions and the mixtures were stored at 4 °C for 16 h.

Urea-ammonium bicarbonate buffer (8 M urea in100 mM ammonium bicarbonate) was added to bovine serum albumin protein standards and *B. cinerea* protease samples to solubilize proteins. Aliquots of solubilized protein (10 µg) were mixed with 10 mM dithiothreitol (1 µL, dithiothreitol; 5 mM final concentration) to reduce sulfide bonds and incubated at room temperature (~ 22 °C) for 1 h. The resulting reduced cysteines were alkylated by the addition of 1 µL 55 mM iodoacetamide in 100 mM ammonium bicarbonate (14 mM final concentration) and incubated in the dark, at room temperature for 20 min. The alkylation reactions were stopped by the addition of 3 µL 10 mM dithiothreitol dissolved in ammonium bicarbonate and the final urea concentration in the samples was reduced below 1 M by the addition of 100 mM ammonium bicarbonate buffer. C_18_ membrane (Thermo-Fisher, Waltham, MA, USA) was conditioned by addition of 50% (v/v) acetonitrile/0.1% (v/v) trifluoroacetic acid, equilibrated with 0.1% (v/v) trifluoroacetic acid, and used to desalt *B. cinerea* peptides. *B. cinerea* peptides were eluted from the membrane with 10 µL of 50% (v/v) acetonitrile iRT peptide buffer. Peptide mixtures were analyzed on a TripleTOF® 6600 + mass spectrometer (SCIEX, Mt Waverley, Australia). Peptides were eluted with either a 9.5-min gradient in the 25-min method (for quality control samples) or a 40-min gradient in the 65-min method (*B. cinerea* protease samples), with mobile phases A and B at a constant flow rate of 300 nL min^−1^. The proportions of solvents A and B were adjusted at specified time-points during the 25- and 65-min methods as follows: (i) 25-min method: 0, 5, 7, 9.5, 10.2, and 20 min corresponding to 95, 60, 10, 10, 95, and 95% of solvent A and (ii) 65-min method: 0, 30, 35, 40, 49, 50, and 60 min corresponding to 98, 60, 35, 10, 10, 98, and 98% of solvent A. Dynamic exclusion was set at 3 or 9 s to account for the difference in chromatographic peak width between the 25- and 60-min methods.

Eluted peptides were subjected to a cyclic data-independent acquisition using even isolation windows SWATH-MS™ acquisition 65-min method (SCIEX, Mt Waverley, Australia), as previously described (Gillet et al. 2012). Specifically, survey scan data (MS) was acquired for 0.08 s followed by MS/MS on all precursors within a selected isolation window in a cyclic manner using an accumulation time of 0.08 s per individual SWATH-MS window. Thirty-six overlapping windows, each 26 m/z units wide, were used to cover the peptide ions in a range of 350–1250 m/z which resulted in the cycle time of 3 s. Fragment ions were recorded in a high sensitivity mode and in a range of 100–1800 m/z. Given the peptide chromatographic peaks were ~ 18-s wide, the above parameters allowed the collection of at least 6 data points for each chromatographic peak to ensure accurate quantitation.

A peptide database was created by downloading canonical and isoform protein sequences for *B. cinerea* in FASTA format from the UniProt website (www.uniprot.org; accessed 19/11/2018). Indexed retention time (iRT) peptides (adapted from Escher et al. (2012); Supplemental Table S1) and common contaminant sequences from the common Repository of Adventitious Proteins, cRAP (http://www.thegpm.org/crap/; provided by Central Analytical Research Facility, Queensland University of Technology, Brisbane, Australia) were added to the file. Spectral data from data-dependent analysis of quality control sample was then analyzed against *B. cinerea* protein database using ProteinPilot software (V5.0, ABSciex, Framingham, MA, USA). A background library of *B. cinerea* peptides was built using Skyline Daily software (MacCoss Lab Software, Seattle, WA, USA), and *B. cinerea* protease samples imported for identification. Any peptide groups with less than 2 peptide matches were removed. The identified list of proteins was exported and compared against the UniProt database (https://www.uniprot.org/). Specifically, the identified UniProt entry name for each protein group was searched again in UniProt, then any similar proteins with 100% identity match from *B. cinerea* were checked for a more descriptive identification. For example, a “putative aspartic protease protein” was identified by the original search but was subsequently identified as Bcap8 with a 100% match in the “similar proteins” section.

## Results

### Evaluating wild-type *B. cinerea* isolates for laccase and protease production

#### Agar plate assays

**Fig. 1:**
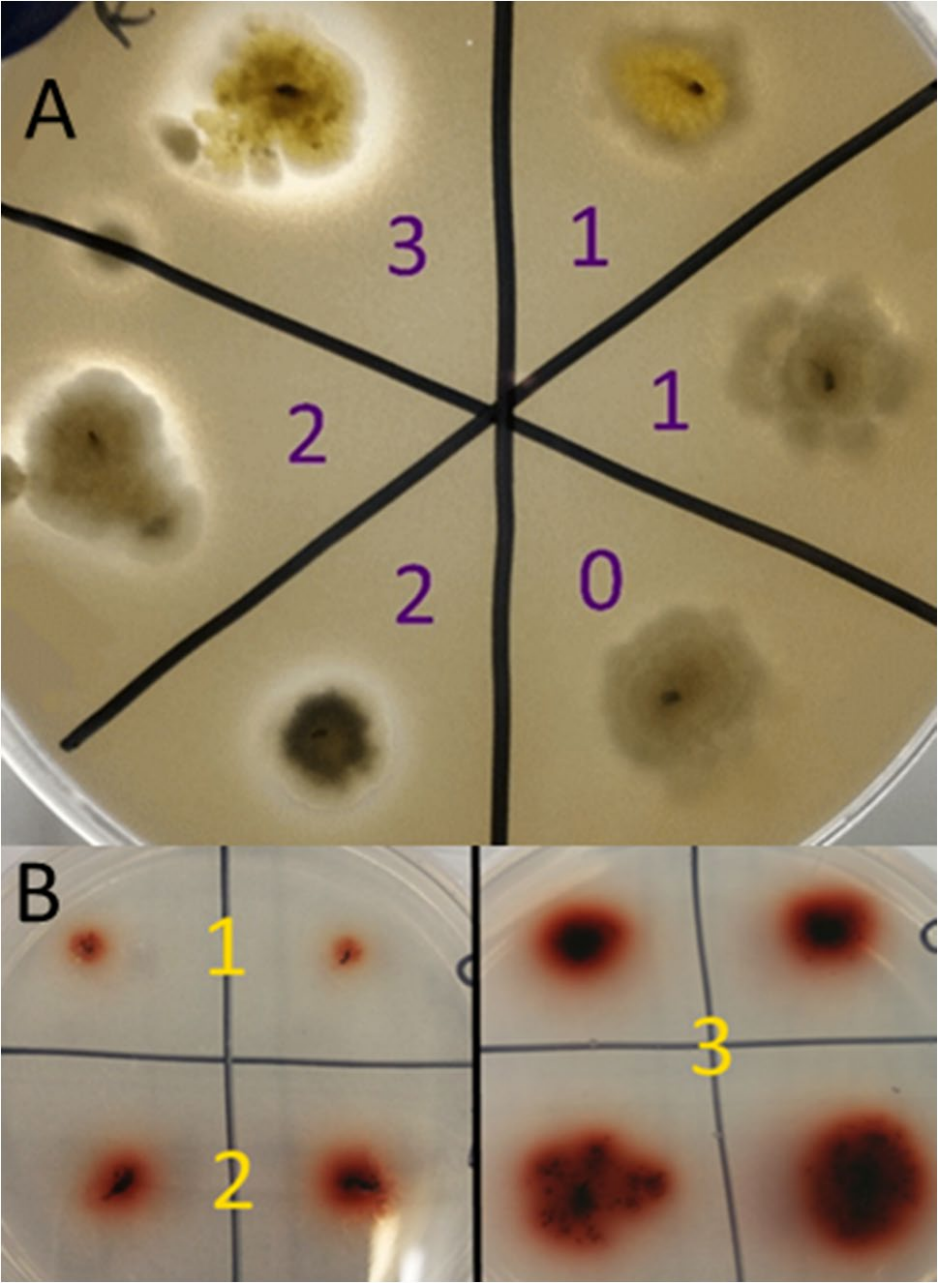
**A** *B. cinerea* isolates growing on skim milk agar plates for semi-quantitative comparison of protease production. Numbers indicate the score allocated to the zone of clearing produced by secretion of proteases from each isolate. **B** *B. cinerea* isolates growing on guaiacol agar plates for semi-quantitative comparison of laccase production. Numbers indicate the score allocated to the coloring of the media produced by oxidation of guaiacol from the secretion of laccase

Eighty-six wild-type isolates of *B. cinerea* were analyzed for protease production on skim milk agar, and for laccase production on guaiacol agar. The scoring system for zones of clearing in skim milk agar, from the cleavage of casein by secreted proteases, provided a semi-quantitative analysis for comparison and down-selection of *B. cinerea* isolates (Fig. 1A). This resulted in the identification of three *B. cinerea* isolates with strong protease production for continuing experimentation; RV05 isolated in Robin Vale, Victoria, and AH42 and AH55 isolated from Adelaide Hills, South Australia. Scoring of the progressive red/brown color change of guaiacol agar during growth of *B. cinerea* isolates also enabled a robust comparison of laccase production between isolates (Fig. 1B), though no isolate that produced both strong levels of proteases and low levels of laccase (an ideal occurrence) was identified.

#### Comparison of protease production in shake flask cultures

The three elite *B. cinerea* isolates identified by skim milk agar assay were cultured in 250-mL shake flasks containing SBF medium. Each experiment was repeated three times, with three biological replicates per experiment. Proteases produced by the isolates were quantified by EnzChek® Protease Assay, identifying *B. cinerea* RV05 as the elite protease producing strain in every experiment (Table 2). Furthermore, *B. cinerea* RV05 produced at least fourfold more protease than *B. cinerea* AH42 or AH55 in all three replicated experiments.

**Table 2:** Results of EnzChek® assay for analysis of protease activity from *B. cinerea* isolates grown in soybean flour medium in 250-mL shake flask culture

*B. cinerea* isolate	Protease (g L^−1^)		
AH42	0.07 ± 0.01	0.12 ± 0.06	0.01 ± 0.02
AH55	0.04 ± 0.02	0.09 ± 0.03	0.01 ± 0.01
RV05	0.31 ± 0.01	0.35 ± 0.08	0.15 ± 0.04

### Optimizing *B. cinerea* protease and laccase production in shake flask cultures

#### Analyzing laccase inhibition by EDTA and CaCl_2_

**Fig. 2:**
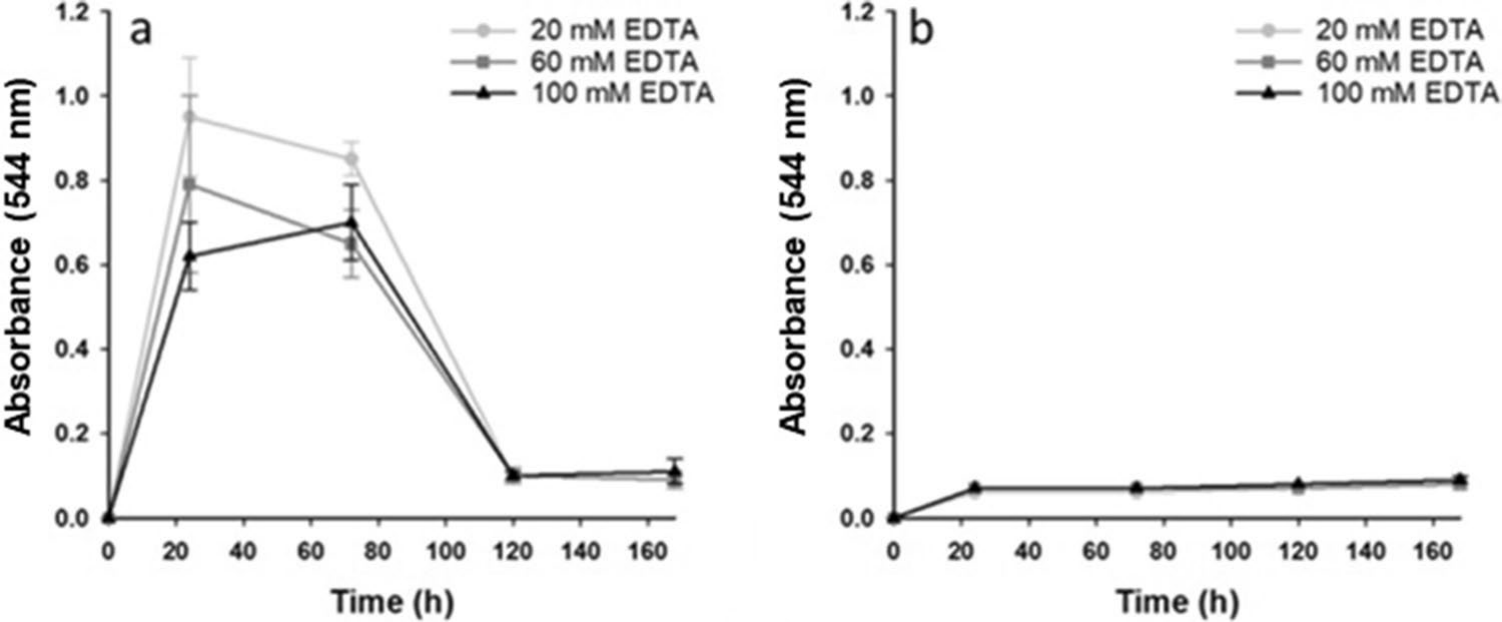
Laccase activity in soybean flour medium plus EDTA from *B. cinerea* isolate RV05, showing the end-point absorbance of guaiacol assay over time. Panel **a** shows results of soybean flour medium in 250-mL shake flasks with malic acid added for pH control, while panel **b** shows results with no malic acid added

Inhibition of laccase production (while maintaining strong protease production) by *B. cinerea* RV05 was analyzed in 250-mL shake flask cultures, by the addition of 20, 60, and 100 mM EDTA, and malic acid for pH reduction. Quantification of laccase production was performed with the guaiacol assay, and results indicate that laccase was produced by *B. cinerea* RV05 at all addition rates of EDTA (Fig. 2a). Protease production was also evident in shake flasks containing EDTA and malic acid, though at lower amounts than controls containing no EDTA (Table 3).

**Table 3:** Maximum protease activity in medium from *B. cinerea* RV05 250-mL shake flask fermentations, analyzed by EnzChek® assay

Description	Additive	Concentration (mM)	Maximum protease (g L^−1^)
EDTA and malic acid	EDTA	0	0.17 ± 0.1
		20	0.04 ± 0.0
		60	0.08 ± 0.0
		100	0.04 ± 0.0
EDTA	EDTA	20	0.00 ± 0.0
		60	0.00 ± 0.0
		100	0.01 ± 0.0
CaCl_2_	CaCl_2_	1.68	0.24 ± 0.0
		72.08	0.30 ± 0.1
		144.16	0.25 ± 0.0

The preparation of SBF media with EDTA and malic acid resulted in a pH of 3 or less after autoclaving, so laccase production by *B. cinerea* isolates in SBF medium with EDTA only (no malic acid) was analyzed. The resulting cultures were at desired pH 3.5, though laccase was still recorded at every sampling point (Fig. 2b). Furthermore, the removal of malic acid from shake flasks containing EDTA greatly reduced protease production from *B. cinerea* RV05 (Table 3), so EDTA was deemed unsuitable for inhibition of laccase production.

**Fig. 3:**
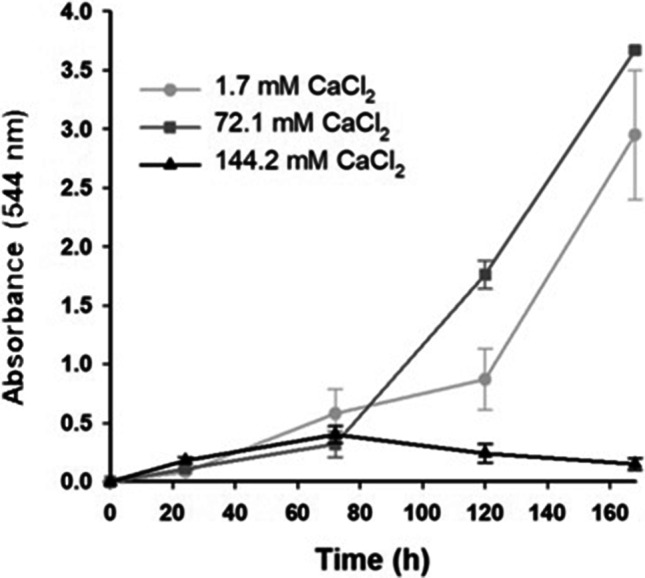
Laccase activity in soybean flour medium plus CaCl_2_ from *B. cinerea* isolate RV05, showing the end-point absorbance of guaiacol assay over time

Inhibition of laccase production by *B. cinerea* RV05 was analyzed in 250-mL shake flask cultures, with the addition of 1.68, 72.08, and 144.16 mM CaCl_2_. Guaiacol assay analysis identified that laccase was produced at every addition rate of CaCl_2_, and at increasing amounts in the shake flasks containing 1.68 and 72.08 mM CaCl_2_ (Fig. 3). Shake flasks containing 144.16 mM CaCl_2_ showed a comparatively reduced rate of laccase production, and protease production remained strong in all shake flasks containing CaCl_2_ (Table 3). However, laccase needed to be completely inhibited for these methods to be successful, so CaCl_2_ was not suitable for inhibition of laccase production in *B. cinerea.*

#### Comparison of elite wild-type *B. cinerea* isolate to laboratory reference strain

A laboratory reference isolate, *B. cinerea* B05.10, was introduced to compare protease production with elite wild-type isolate, *B. cinerea* RV05. EnzChek® Protease Assay was used to analyze protease production in SBF medium in three separate experiments (SBF 1, 2, and 3), with three biological replicates per experiment. In every experiment, *B. cinerea* RV05 produced higher maximum protease amounts than *B. cinerea* B05.10 (Table 4), indicating that *B. cinerea* RV05 was the elite strain for protease production in 250-mL shake flask cultures.

**Table 4:** Maximum protease activity in medium from 250-mL fermentation of *B. cinerea* RV05 and B05.10, analyzed by EnzChek® assay

Experiment	*B. cinerea* RV05 (g L^−1^)	*B. cinerea* B05.10 (g L^−1^)
SBF 1^1^	0.17 ± 0.01	0.05 ± 0.01
SBF 2	0.19 ± 0.02	0.05 ± 0.01
SBF 3	0.29 ± 0.06	0.10 ± 0.02
Lactic acid 50 mM	0.15 ± 0.03	0.10 ± 0.02
Lactic acid 100 mM	0.17 ± 0.03	0.12 ± 0.02
Malic acid 50 mM	0.12 ± 0.06	0.12 ± 0.06

^1^The data presented for SBF 1, 2, and 3 were from experimental replicates, while the data presented from SBF media containing added lactic and malic acid were from a single experiment and three biological replicates

#### Optimizing liquid medium to reduce glucan production

When analyzing the inhibition of laccase by EDTA, addition of malic acid to SBF shake flask cultures resulted in a more viscous culture medium when compared to the shake flasks to which no malic acid was added. Reduction in glucan production was investigated by adding lactic acid (another acid produced in the winemaking process), while maintaining strong protease production from both *B. cinerea* RV05 and B05.10. A significant reduction in the viscosity of culture medium was observed with 50 and 100 mM lactic acid added to SBF medium, assessed by the ease of sample collection and the movement of culture medium when swirled in a flask. Maximum protease production from *B. cinerea* RV05 was observed in culture medium containing 100 mM lactic acid in comparison to malic acid (Table 4), though little difference was observed between acids for *B. cinerea* B05.10. Based on the strong production of protease and reduced culture viscosity in shake flasks, 100 mM lactic acid was identified as the superior acid for pH reduction in *B. cinerea* shake flask cultures.

### Scaling and optimizing protease production from *B. cinerea* RV05 in 1-L bioreactors

Protease production from *B. cinerea* isolates was scaled from 250-mL shake flasks to 1-L bioreactor vessels. *B. cinerea* RV05 was used for initial testing due to its strong protease production in small-scale experiments. Fermentation 1 was performed with conditions analogous to small-scale experiments, i.e., no pH control. *B. cinerea* RV05 produced a maximum of 0.83 ± 0.1 g L^−1^ protease (Table 5), a marked increase from maximum protease amounts observed in small-scale experiments.

**Table 5:** Maximum protease activity in media from 1-L *B. cinerea* RV05 and B05.10 fermentation, analyzed by EnzChek® assay

Description	*B. cinerea* strain	Protease (g L^−1^)
Fermentation 1	RV05	0.83 ± 0.1
Fermentation 2	RV05	0.52 ± 0.0
Fermentation 3	RV05	0.78 ± 0.0
Fermentation 4	RV05	3.04 ± 0.2
Fermentation B1	B05.10	2.60 ± 0.3
Fermentation B2	B05.10	1.75 ± 0.0
Fermentation B3	B05.10	3.60 ± 0.9

Fermentation 2 was operated with pH control, and 2 N H_2_SO_4_ and 3 N NaOH to maintain the pH at 3.5. Protease production from *B. cinerea* RV05 was again greater than the amounts observed in small-scale experiments. However, the continual addition of acid and base to maintain a pH of 3.5 resulted in a volume increase from 0.8 to over 1.0 L by the end of the fermentation. Fermentation 3 was maintained at a pH range of 3.5 to 4.0, with only the acid pump activated to maintain the pH below 4, and lactic acid for pH reduction. The resulting maximum protease amount was similar to that observed in fermentation 1, at 0.78 ± 0.0 g L^−1^. The culture medium also became increasingly viscous as the fermentation continued, from increased glucan production from *B. cinerea*.

Fermentation 4 with *B. cinerea* RV05 was performed with H_2_SO_4_ for pH control, and the acid pump alone used for pH maintenance. The removal of lactic acid resulted in a marked decrease in the viscosity of the culture medium, and the highest maximum protease production from *B. cinerea* RV05 was recorded, at 3.04 ± 0.2 g L^−1^.

While optimizing conditions for 1-L bioreactors, a fault with a pH probe resulted in a drastic pH drop in the culture medium from over addition of acid, resulting in a culture medium below pH 1.5. The pH was corrected, and the fermentation run to completion, to observe the effect of very low pH on the growth of *B. cinerea* RV05. EnzChek® assay analysis indicated that protease levels had been reduced, though not completely removed by the strong pH drop (*data not shown*). SDS-PAGE analysis revealed that the ~ 78-kDa band presumptively associated with laccase was not visible in samples collected in the hours following the drastic pH drop (Supplemental Fig. S1), suggesting that low pH treatment of *B. cinerea* culture medium could inactivate laccase activity while maintaining some protease activity.

### Analysis of protease production from *B. cinerea* B05.10 in 1-L bioreactors

**Fig. 4:**
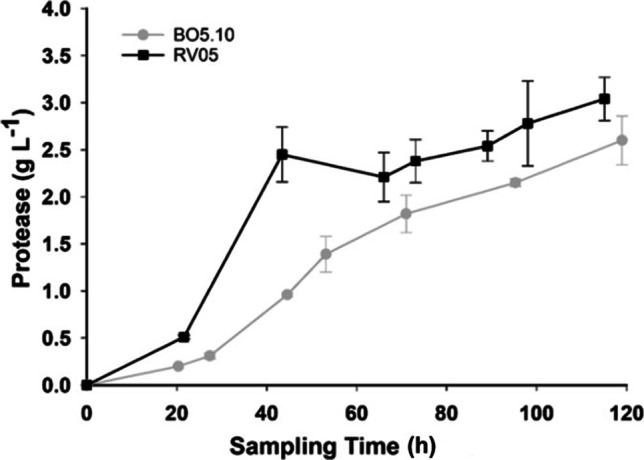
Comparison of EnzChek® assay results for quantitation of protease production from *B. cinerea* RV05 and B05.10 cultured in soybean flour medium in 1-L bioreactors

Protease production from *B. cinerea* B05.10 in 1-L bioreactors was also evaluated since it is an isolate used by many researchers worldwide, and successful production of protease with this isolate would increase the opportunity for reproduction of results in other laboratories. Fermentations were performed with the optimized conditions established with *B. cinerea* RV05 and compared with results from fermentation 4. Figure 4 shows the recorded protease amounts by EnzChek® assay during fermentation for both *B. cinerea* RV05 and B05.10. Single factor ANOVA analysis of protease recorded at all time points revealed that there was no statistical difference in protease production between isolates (*p-*value = 0.14). Single factor ANOVA of the maximum protease amount recorded at the end of fermentation indicated no statistical difference between isolates (*p*-value = 0.11). Two further fermentations were performed in parallel to confirm strong protease production from *B. cinerea* B05.10 in bioreactors. In replicate fermentations B2 and B3, the strong protease production from *B. cinerea* B05.10 continued, including the largest amount of protease recorded by EnzChek® assay in 1-L fermentations, of 3.60 ± 0.9 g L^−1^ (Table 5).

### Inactivation of laccase with pH reduction

**Fig. 5:**
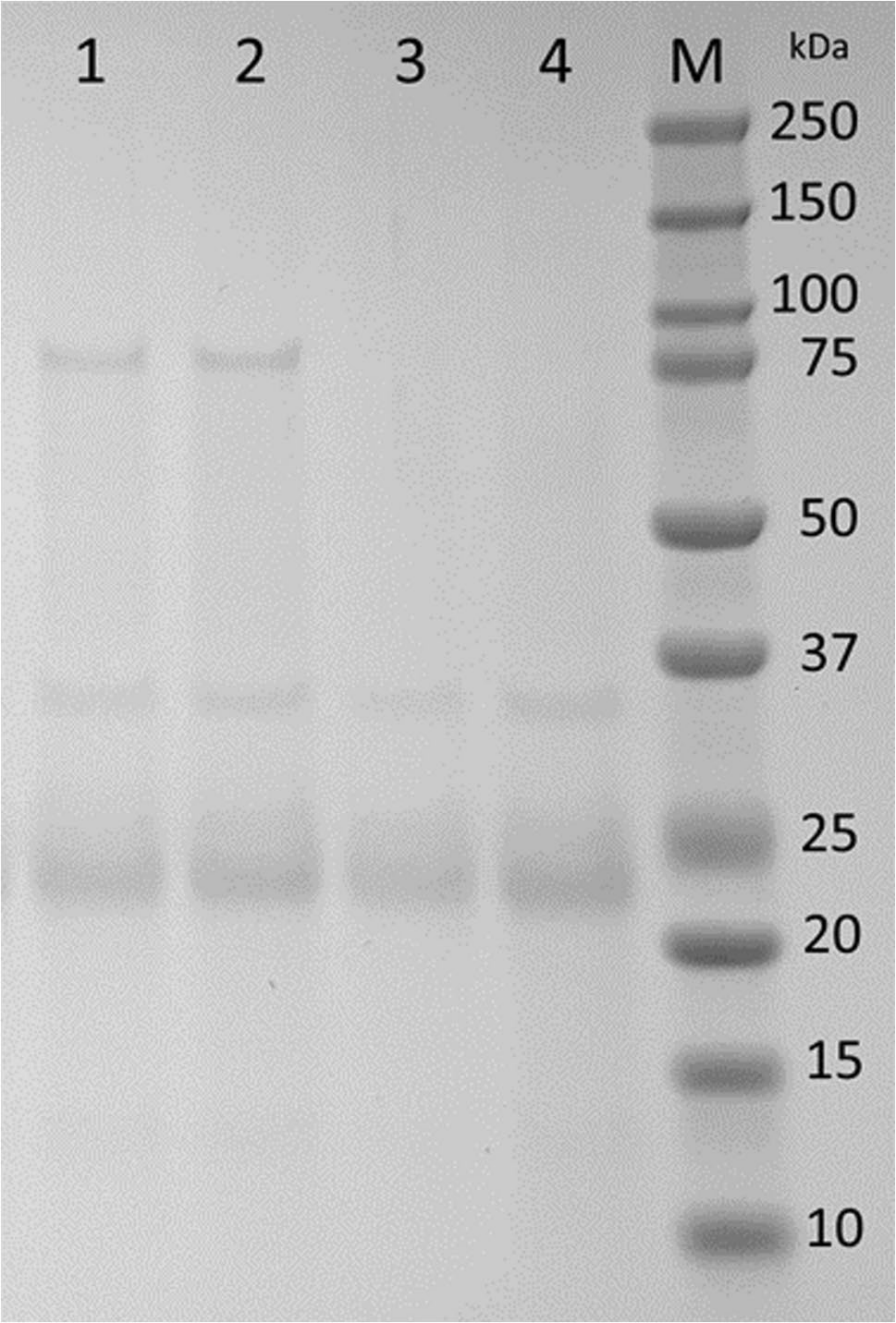
Resolution of protease containing supernatant from *B. cinerea* fermentation using SDS-PAGE. Duplicate samples of *B. cinerea* supernatant before (lanes 1 and 2) and after (lanes 3 and 4) low pH treatment were resolved on a 4–12% gradient gel. Lane M contains Bio-Rad Precision Plus Protein Dual Color Standard and the molecular masses (kDa) of the markers are indicated on the right

The inactivation of laccase (while maintaining some protease activity) was analyzed by reducing aliquots of *B. cinerea* protease containing supernatant to pH 1.5, then returning to pH 4.0 after 1 h. Resolution of the supernatant before and after the pH drop on SDS-PAGE indicates that the ~ 75-kDa band associated with laccase was not visible following pH treatment, while the ~ 35-kDa band associated with abundant *B. cinerea* protease BcAP8 was still visible (Fig. 5). Bands at ~ 23 kDa represent unidentified *B. cinerea* proteins that were also not degraded at pH 1.5. Quantification of protease activity by EnzChek® protease assay (in triplicate) indicated that after pH reduction, protease activity had been reduced from 2.02 ± 0.24 g L^−1^ to 1.01 ± 0.20 g L^−1^.

The re-activation of laccase when added to grape juice was analyzed by guaiacol assay, with overnight incubation. Positive controls and *B. cinerea* supernatant samples before pH reduction both measured end-point absorbance above the maximum (4.32 ± 0.0), while the protease supernatant reduced to pH 1.5 had a negative reading (− 0.06 ± 0.0) indicating that laccase was not re-activated when added to grape juice.

### Analyzing ammonium sulfate precipitation of *B. cinerea* secreted proteins

Precipitation of proteases from the supernatant was analyzed with ammonium sulfate at 40, 60, and 80% (w/v) saturation. EnzChek® assay results of 10-mL experiments indicated that 0.8% of total active protease was collected at 40% ammonium sulfate saturation, 5.9% at 60% saturation, and 64.41% at 80% saturation. Ammonium sulfate precipitation at 80% saturation with 100-mL volumes resulted in average protease yields of 66.63 ± 6.2% in triplicate analyses.

### Identification of *B. cinerea* secreted proteins by mass spectrometry

Peptides from *B. cinerea* proteins precipitated by ammonium sulfate were analyzed by mass spectrometry with SWATH acquisition and identified by comparison to a library of *B. cinerea* peptides. A total of 41 protein groups were identified in the *B. cinerea* protease mixture (Supplemental Table S2). The most abundant protein identified was glucoamylase, followed by aspartic protease BcAP8, serine peptidase, and glycoside hydrolase (peptide identifications, *n* ≥ 11), all enzymes relating to cell wall degradation and virulence of *B. cinerea* (González-Fernández et al. 2014). Proteins with between 6 and 9 peptide identifications include sedolisin (serine protease), laccase-2, tripeptidyl-peptidase, carboxylic ester hydrolase, and glycoside hydrolase.

## Discussion

Screening *B. cinerea* isolates for protease production on skim milk agar proved a reliable and simple method for identifying isolates that secreted proteolytic enzymes at pH 3.5. Skim milk medium has been used to reliably identify proteolytic activity from *Cladosporium* and *Trichoderma* species amongst a cohort of filamentous fungi, using a similar rating to compare isolates (Nwadiaro et al. 2015). Guaiacol agar has also been used to identify filamentous fungi with laccase producing capacity, such as *Trichoderma harzianum* (Abd El Monssef et al. 2016), allowing simple identification of elite strains for further experimentation. The identification of a *B. cinerea* isolate that had simultaneously strong protease production and low laccase production was not achieved, though was also not likely as overall protein secretion has been linked to the virulence (and protease production) of a *B. cinerea* isolate (Fernández-Acero et al. 2007). Alternative carbon sources may have changed the secretion of protease or laccase from *B. cinerea*, which has been known to alter protein expression in relation to its environment (Liñeiro et al. 2016). More opportunities for modifying culture medium to illicit the desired response from *B. cinerea* were presented in small-scale liquid medium experiments.

Culturing the down selected *B. cinerea* isolates in SBF medium, with quantification of proteases by EnzChek® assay, proved a reliable method for identifying *B. cinerea* isolates with strong protease production. Soybean flour is a robust, cheap, and readily available nitrogen source for culturing fungi, which has been evaluated as a N source in cultures of various filamentous fungi to produce statins from *Monascus* and *Aspergillus* (Manzoni et al. 1999), proteases from thermophilic fungi (Macchione et al. 2008), and has been optimized for bioactive compound production in solid-state fermentation (Handa et al. 2019). Soybean protein (flour) was also identified as a good inducer of proteases production from *B. cinerea* when evaluating low-cost raw materials to produce alkaline proteases (Abidi et al. 2008). However, a more environmentally friendly and sustainable option for culturing *B. cinerea* isolates could be found by analyzing winery waste products as C and N sources. Large amounts of waste are produced at all stages of winemaking, including the remains from destemming and pressing grape berries, and sediment from clarification steps (Devesa-Rey et al. 2011). Grape vines and berries are a common target for *B. cinerea* (Ciliberti et al. 2016; Cotoras and Silva 2005; Dewey et al. 2008), so analyzing these waste products as a component of culture medium for the production of proteases from *B. cinerea* would be an ideal target for further investigation.

The inhibition of laccase in shake flask cultures was not achieved. Despite reports that concentrations of EDTA as low as 12 mM had resulted in an absence of laccase in *B. cinerea* culture medium (Bar-Nun et al. 1988), laccase production was detected at every concentration of EDTA added to SBF medium. There was less laccase detected when no malic acid was used with EDTA; however, this coincided with almost no protease production. This result indicates that malic acid was being used as a nutrient source when added to the soybean flour medium for pH control. CaCl_2_ has been shown to reduce laccase when added to culture medium (Sansone et al. 2011), but strong laccase production from *B. cinerea* RV05 was observed when 1.68 and 72.08 mM CaCl_2_ were added to shake flasks. While a comparatively reduced amount of laccase was observed in the shake flasks containing SBF and 144.16 mM CaCl_2_, even small amounts of laccase can have an adverse effect on the quality of wine (Zimdars et al. 2017), so complete removal or inhibition of laccase from *B. cinerea* isolates is required.

In shake flasks containing SBF medium, *B. cinerea* RV05 regularly produced more protease than reference strain *B. cinerea* B05.10. When malic acid was replaced with lactic acid for pH control, B05.10 produced more protease than previously, but still not as much as RV05. Other studies have identified *B. cinerea* B05.10 as a strong protein producer in comparison to two other wild-type *B. cinerea* isolates from grapes (Quijada‐Morin et al. 2018), which implies that two strong protease producing strains of *B. cinerea* have been identified in this work.

Lactic acid proved a superior choice for pH control of *B. cinerea* culture medium than malic acid. Both malic and lactic acids are formed in the winemaking process (Wibowo et al. 1985), so are ideally suited for pH reduction in this work. Because the chemical structures of these acids vary, in that malic acid has two carboxyl groups and lactic acid only one, both the same concentration and double concentration of lactic acid compared to malic acid were tested. This allowed a direct comparison to both the equivalent molarity and buffering capacity of each acid. Analysis of carbon sources metabolized by *B. cinerea* using Biolog FF Microplates (Wang et al. 2016) found that both D- and L-malic acid were moderately metabolized by wild *B. cinerea* isolates, while L-lactic acid was not metabolized. The reduction in glucan production when lactic acid was used to control media pH was likely due to the inability of *B. cinerea* to metabolize the acid. Without additional substrate to feed the carbohydrate-rich glucan (Stahmann et al. 1995), the medium did not become as viscous as with malic acid, which increased the ease of sampling and made down-stream processing of samples more simple and efficient.

Scaling protease production from shake flask volumes to 1-L bioreactors presented the opportunity to increase both the volume and concentration of proteases produced by the *B. cinerea* isolates. The protease results from fermentation 1 (with no pH control) resulted in a 2 to 3 × increase in protease secretion from RV05 in SBF medium. Subsequent fermentations identified that *B. cinerea* requires only acid addition to maintain the culture medium below pH 4. This has been observed in other research, where the fungus has been shown to adjust the pH of its environment to suit growth requirements (Billon‐Grand et al. 2012; Manteau et al. 2003; Verhoeff et al. 1988).

Lactic acid was also analyzed for pH reduction in 1-L fermentations, as this acid had worked well in small-scale experiments. However, in fermentation 3, the culture medium turned very viscous after several days of culturing, with the return of lactic acid for pH control being the only chemical change introduced. This is curious since lactic acid had resulted in reduced culture viscosity in small-scale experiments, and *B. cinerea* has been shown not to metabolize lactic acid (Wang et al. 2016). Sulfuric acid was returned for pH control in fermentation 4 (Eppendorf 2012), and resulted in less viscous culture medium in 1-L *B. cinerea* fermentations. With this final change, protease levels were the highest recorded with over 3 g L^−1^ produced by the end of the fermentation, for a tenfold increase on the maximum protease amounts produced by *B. cinerea* RV05 in small-scale experiments.

The difference in quantified protease levels in the more viscous fermentation with lactic acid could be due to the protease and other metabolites produced by *B. cinerea* being trapped inside the glucan rich biomass, instead of dissolving in the small amount of free-flowing medium in the vessel. This sequestering of metabolites in *B. cinerea* culture medium has been observed in previous studies, when β-1,3-glucanase was added to culture medium to reduce the viscosity, resulting in an observed increase in laccase activity (Gil-ad et al. 2001). However, this was coupled with an increase in laccase activity; not an ideal outcome when laccase activity is not desired.

*B. cinerea* B05.10 was ultimately selected as the strain to continue scale up work, and steadily produced high levels of protease across three 1-L fermentations. *B. cinerea* B05.10 has been used in many different experimental contexts, from transcriptome profiling (Srivastava et al. 2020) to morphological growth analyses (Zhou et al. 2018), and has had its entire genome sequenced (Amselem et al. 2011; Van Kan et al. 2016). *B. cinerea* B05.10 has also been used in labs across the world, as it has maintained sterility and virility with a reduced nuclear number (Tudzynski and Siewers 2007). *B. cinerea* B05.10 showed continued strong protease production in 1-L bioreactors, also showing a greater than 10 times increase from protease levels observed in shake flasks.

A major aim in this work was to inactivate or remove laccase from the protease in culture medium. The accidental, drastic reduction in pH during an early 1-L fermentation provided insight that *B. cinerea* laccase could be denatured by decreasing the medium pH close to pH 1. Indeed, it has been discovered that *B. cinerea* laccase will be irreversibly denatured below pH 2.5 (Dubernet et al. 1977). Analysis of *B. cinerea* expressed proteins by SDS-PAGE enabled simple conformation of the absence of laccase from the analyzed sample, and is a method used for analysis of *B. cinerea* proteins by other researchers (Marchal et al. 2020; Shah et al. 2009).

The possibility of re-activation of laccase was assessed by incubating samples overnight at room temperature, with guaiacol as the indicator. The extended incubation period allowed for any laccase present to be re-activated and detected by a color change of guaiacol from clear to deep red brown. No laccase activity was detected in any of the protease samples after addition to grape juice, confirming that laccase had been inactivated in the *B. cinerea* culture medium.

The proteins present in the ammonium sulfate fraction from *B. cinerea* were identified by SWATH-MS. Since the protease preparation was reduced to pH 1.5 prior to ammonium sulfate precipitation, it is not certain that any of the proteases identified by SWATH-MS were able to maintain enzymatic activity and contribute to the overall protease activity recorded in the final product. However, the EnzChek® protease assay used for quantifying protease activity in the samples will detect metallo, serine, sulfhydryl, and acid proteases (BcAP8) (Probes 2004). Therefore, while it is likely that most of the protease activity detected in the *B. cinerea* protease mixture was BcAP8, as it is the most abundant protein in the *B. cinerea* secretome and in this sample (Espino et al. 2010; Ten Have et al. 2010) and known to be active at low pH, it is possible that serine and metallo-proteases also added to the protease activity detected by EnzChek® assay.

Previous studies of the *B. cinerea* proteome have identified differences in protein expression and secretion with differing substrates, whether in liquid or solid cultures (Shah et al. 2009), and also at different pH (Li et al. 2012). The *B. cinerea* culture from which these proteins were collected was maintained at pH 4, which has been shown to induce secretion of proteases that counter the host’s pathogenesis response rather than proteases aimed at cell wall degradation (González-Fernández et al. 2015). While the most abundant protein identified in the *B. cinerea* protease mixture was glucoamylase, a plant cell wall degrading enzyme (Li et al. 2020), which has been identified in high abundance from *B. cinerea* B05.10 by other researchers (González-Fernández et al. 2014), most of the proteins identified by 5 or more peptides were proteases. Finally, the identification of laccase-2 in the protease cocktail is not concerning but highlights that 80% ammonium sulfate precipitation collected all the proteins and protein fragments (whether active or inactivated) present in the *B. cinerea* protease mixture.

Through the screening, down selection, and optimization of culture medium, two *B. cinerea* strains with strong protease production have been identified. Scale up of production from 250-mL shake flasks to 1-L bioreactors increased protease yield over 10 times. The precipitation of proteases with 80% ammonium sulfate resulted in the preparation of a simple protease cocktail with the potential to degrade wine-haze forming proteins and reduce the use of bentonite in the winemaking process.

## Supplementary Information

Below is the link to the electronic supplementary material.

Supplementary file1 (PDF 362 KB)

## Data Availability

The datasets generated during and/or analyzed during the current study are available from the corresponding author on reasonable request.
