# Supplementary material for: Development of simple, scalable protease production from Botrytis cinerea
Source: Appl Microbiol Biotechnol. 2022 Feb 16;106(5-6):2219–33. doi: 10.1007/s00253-022-11817-1 (PMC8930891; doi:10.1007/s00253-022-11817-1)
Supplement: Supplementary file 1 — Supplementary file1 (PDF 362 KB) [file 253_2022_11817_MOESM1_ESM.pdf]

Applied Microbiology and Biotechnology

Development of simple, scalable protease production from *Botrytis cinerea* for wine stabilization

Rachel A. Self [1,2]*, Mark D. Harrison [1,2], Valentino S. (Junior) Te'o [2], and Steve Van Sluyter [3]

[1] Centre for Agriculture and the Bioeconomy, Queensland University of Technology, Brisbane, Queensland 4000, Australia

[2] School of Biology and Environmental Science, Queensland University of Technology, Brisbane, Queensland 4000, Australia

[3] Department of Biological Sciences, Macquarie University, Sydney, New South Wales, 2109, Australia

*Corresponding author: Rachel Self, +61 428 769 351, rachel.self@qut.edu.au

Supplementary Data

Table S1. Amino acid sequences of iRT peptides. Amino acid sequence of iRT protein and elution order of subsequent peptides used during protein identification by SWATH

**iRT protein**	
LGGNEQVTRGAGSSEPVTGLDAKVEATFGVDESNAKYILAGVENSKTPVISGGPYEYRTPVITGAPY EYRDGLDAASYYAPVRADVTPADFSEWSKGTFIIDPGGVIRGTFIIDPAAVIRLFLQFGAQGSPFLK	
**List of iRT peptides in elution order:**	
1	LGGNEQVTR
2	GAGSSEPVTGLDAK
3	VEATFGVDESNAK
4	YILAGVENSK
5	TPVISGGPYEYR
6	TPVITGAPYEYR
7	DGLDAASYYAPVR
8	ADVTPADFSEWSK
9	GTFIIDPGGVIR
10	GTFIIDPAAVIR
11	LFLQFGAQGSPFLK

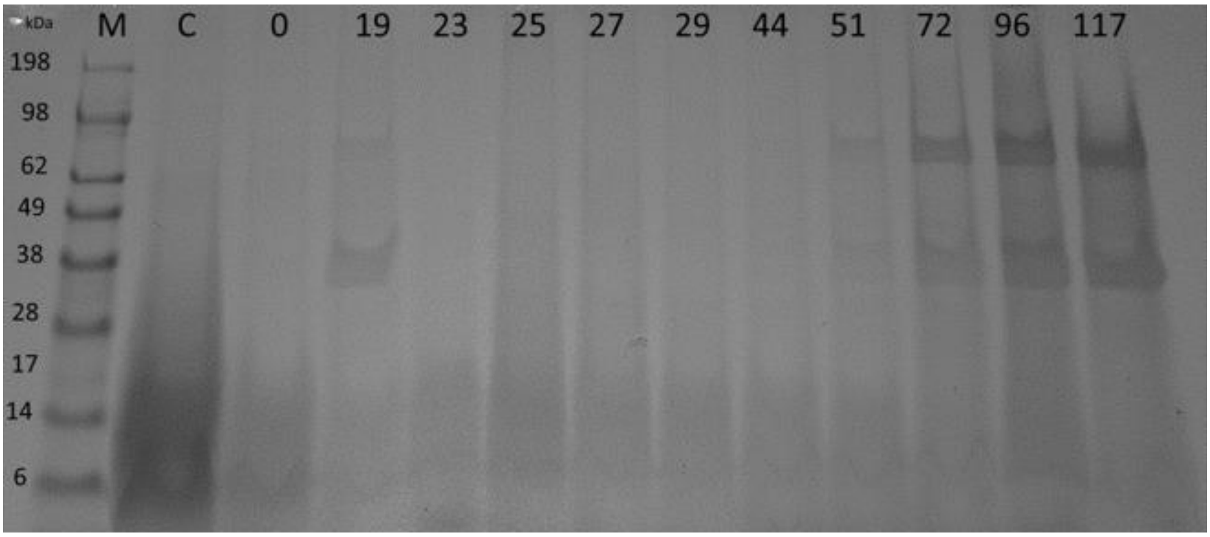

Fig S1 Resolution of proteins in media from *B. cinerea* fermentation using SDS-PAGE. Supernatant samples were collected at time points indicated by the numbers (h) at the top of the lane and were resolved on a 4-12% gradient gel. Multiple samples were collected between 19 and 44 h, as the pH of the fermentation had been dropped below pH 1.5 by a pH probe malfunction. Lane M contains SeeBlue™ Plus2 Pre-stained Protein Standard, lane C contains fermentation medium before inoculation (control) and the molecular masses (kDa) of the markers are indicated on the left

Table S2. Table of *Botrytis cinerea* proteins identified from spectral library in Skyline Daily

UniProt Protein ID	Protein Group Name	No. peptides identified	Organism	Protein ID Original Match (Skyline)
A0A384JFR0	Glucoamylase	24	*Botrytis cinerea*	tr|A0A384JFR0|A0A384JFR0_BOTFB Uncharacterized protein OS=*Botryotinia fuckeliana* (strain B05.10) OX=332648 GN=BCIN_04g04190 PE=4 SV=1
M7UEM4	Bcap8	17	*Botrytis cinerea*	tr|M7UEM4|M7UEM4_BOTF1 Putative aspartic protease protein OS=*Botryotinia fuckeliana* (strain BcDW1) OX=1290391 GN=BcDW1_9462 PE=3 SV=1
G2YQJ4	Putative serine peptidase	12	*Botrytis cinerea*	tr|M7U3E8|M7U3E8_BOTF1 Putative serine protein OS=*Botryotinia fuckeliana* (strain BcDW1) OX=1290391 GN=BcDW1_3285 PE=4 SV=1
M7UN92	Glycoside hydrolase family 17 protein / Putative gpi-anchored cell wall beta endoglucanase protein	11	*Botrytis cinerea*	tr|M7UN92|M7UN92_BOTF1 Putative gpi-anchored cell wall beta endoglucanase protein OS=*Botryotinia fuckeliana* (strain BcDW1) OX=1290391 GN=BcDW1_2966 PE=4 SV=1
G2Y3I7	Sedolisin (serine protease)	9	*Botrytis cinerea*	tr|M7U283|M7U283_BOTF1 Putative subtilisin-like protein OS=Botryotinia fuckeliana (strain BcDW1) OX=1290391 GN=BcDW1_3686 PE=4 SV=1
LAC2	Laccase-2	7	*Botrytis cinerea*	sp|Q96WM9|LAC2_BOTFU Laccase-2 OS=*Botryotinia fuckeliana* OX=40559 GN=lcc2 PE=2 SV=1
M7TQY0	Putative tripeptidyl-peptidase 1 protein	7	*Botrytis cinerea*	tr|M7TQY0|M7TQY0_BOTF1 Putative tripeptidyl-peptidase 1 protein OS=*Botryotinia fuckeliana* (strain BcDW1) OX=1290391 GN=BcDW1_7820 PE=4 SV=1
M7TR26	Carboxylic ester hydrolase	6	*Botrytis cinerea*	tr|M7TR26|M7TR26_BOTF1 Carboxylic ester hydrolase OS=*Botryotinia fuckeliana* (strain BcDW1) OX=1290391 GN=BcDW1_5286 PE=3 SV=1
M7THQ2	Putative glycoside hydrolase family 55 protein	6	*Botrytis cinerea*	tr|M7THQ2|M7THQ2_BOTF1 Putative glycoside hydrolase family 55 protein OS=*Botryotinia fuckeliana* (strain BcDW1) OX=1290391 GN=BcDW1_8368 PE=4 SV=1
A0A384K566	Bcser8 / Putative tripeptidyl peptidase protein	5	*Botrytis cinerea*	tr|A0A384K566|A0A384K566_BOTFB Bcser8 OS=*Botryotinia fuckeliana* (strain B05.10) OX=332648 GN=Bcser8 PE=4 SV=1
M7USX5	Glucanase	5	*Botrytis cinerea*	tr|M7USX5|M7USX5_BOTF1 Glucanase OS=*Botryotinia fuckeliana* (strain BcDW1) OX=1290391 GN=BcDW1_1287 PE=3 SV=1
M7UGX3	Glycosidase	5	*Botrytis cinerea*	tr|M7UGX3|M7UGX3_BOTF1 Putative glycoside hydrolase family 16 protein OS=*Botryotinia fuckeliana* (strain BcDW1) OX=1290391 GN=BcDW1_5218 PE=4 SV=1
M7UP80	Glycoside hydrolase family 93 protein / Putative bnr asp-box repeat domain protein	5	*Botrytis cinerea*	tr|M7UP80|M7UP80_BOTF1 Putative bnr asp-box repeat domain protein OS=*Botryotinia fuckeliana* (strain BcDW1) OX=1290391 GN=BcDW1_2586 PE=4 SV=1

M7UZB2	Carboxypeptidase	5	*Botrytis cinerea*	tr|M7UZB2|M7UZB2_BOTF1 Carboxypeptidase OS=*Botryotinia fuckeliana* (strain BcDW1) OX=1290391 GN=BcDW1_2086 PE=3 SV=1
M7TJF2	1,3-beta-glucanosyltransferase	4	*Botrytis cinerea*	tr|M7TJF2|M7TJF2_BOTF1 1,3-beta-glucanosyltransferase OS=*Botryotinia fuckeliana* (strain BcDW1) OX=1290391 GN=BcDW1_7721 PE=3 SV=1
M7TIW0	Uncharacterized protein	4	*Botrytis cinerea*	tr|M7TIW0|M7TIW0_BOTF1 Uncharacterized protein OS=*Botryotinia fuckeliana* (strain BcDW1) OX=1290391 GN=BcDW1_10395 PE=4 SV=1
Q9HF87	Aspartic proteinase (Bcap1)	3	*Botrytis cinerea*	tr|Q9HF87|Q9HF87_BOTFU Aspartic proteinase OS=*Botryotinia fuckeliana* OX=40559 GN=Bcap1 PE=3 SV=1
M7TMG9	Putative tripeptidyl peptidase protein	3	*Botrytis cinerea*	tr|M7TMG9|M7TMG9_BOTF1 Putative tripeptidyl peptidase protein OS=*Botryotinia fuckeliana* (strain BcDW1) OX=1290391 GN=BcDW1_8798 PE=4 SV=1
M7TD64	Arabinan endo-1,5-alpha-L-arabinosidase	3	*Botrytis cinerea*	tr|M7TD64|M7TD64_BOTF1 Arabinan endo-1,5-alpha-L-arabinosidase OS=*Botryotinia fuckeliana* (strain BcDW1) OX=1290391 GN=BcDW1_9995 PE=3 SV=1
A7X4J2	Polygalacturonase 1	3	*Botrytis cinerea*	tr|A7X4J2|A7X4J2_BOTFU Polygalacturonase 1 (Fragment) OS=*Botryotinia fuckeliana* OX=40559 GN=PG1 PE=3 SV=1
M7TZ84	Putative glycoside hydrolase family 43 protein	3	*Botrytis cinerea*	tr|M7TZ84|M7TZ84_BOTF1 Putative glycoside hydrolase family 43 protein OS=*Botryotinia fuckeliana* (strain BcDW1) OX=1290391 GN=BcDW1_4740 PE=4 SV=1
A0A384J8H1	Glycoside hydrolase family 13 protein	3	*Botrytis cinerea*	tr|A0A384J8H1|A0A384J8H1_BOTFB Uncharacterized protein OS=*Botryotinia fuckeliana* (strain B05.10) OX=332648 GN=BCIN_02g01420 PE=4 SV=1
M7UXY9	Glycoside hydrolase family 54 protein / Putative fungal alpha-l-protein	3	*Botrytis cinerea*	tr|M7UXY9|M7UXY9_BOTF1 Putative fungal alpha-l-protein OS=*Botryotinia fuckeliana* (strain BcDW1) OX=1290391 GN=BcDW1_2562 PE=4 SV=1
M7TT59	Carboxylic ester hydrolase	3	*Botrytis cinerea*	tr|M7TT59|M7TT59_BOTF1 Carboxylic ester hydrolase OS=*Botryotinia fuckeliana* (strain BcDW1) OX=1290391 GN=BcDW1_6999 PE=3 SV=1
Q8X116	Pectinesterase	3	*Botrytis cinerea*	tr|Q8X116|Q8X116_BOTFU Pectinesterase OS=*Botryotinia fuckeliana* OX=40559 GN=bcpme2 PE=4 SV=1
M7TSP5	Uncharacterized protein	3	*Botrytis cinerea*	tr|M7TSP5|M7TSP5_BOTF1 Uncharacterized protein OS=*Botryotinia fuckeliana* (strain BcDW1) OX=1290391 GN=BcDW1_7069 PE=4 SV=1
M7TKZ8	Putative acid phosphatase protein	2	*Botrytis cinerea*	tr|M7TKZ8|M7TKZ8_BOTF1 Putative acid phosphatase protein OS=*Botryotinia fuckeliana* (strain BcDW1) OX=1290391 GN=BcDW1_9366 PE=4 SV=1
M7U9N2	Putative metalloproteinase protein	2	*Botrytis cinerea*	tr|M7U9N2|M7U9N2_BOTF1 Neutral protease 2 OS=*Botryotinia fuckeliana* (strain BcDW1) OX=1290391 GN=BcDW1_8058 PE=3 SV=1
A0A384J9H4	Putative glycoside hydrolase family 1 protein	2	*Botrytis cinerea*	tr|A0A384J9H4|A0A384J9H4_BOTFB Uncharacterized protein OS=*Botryotinia fuckeliana* (strain B05.10) OX=332648 GN=BCIN_02g03090 PE=4 SV=1

M7TNY7	Putative glycoside hydrolase family 55 protein	2	*Botrytis cinerea*	tr|M7TNY7|M7TNY7_BOTF1 Putative glycoside hydrolase family 55 protein OS=*Botryotinia fuckeliana* (strain BcDW1) OX=1290391 GN=BcDW1_8537 PE=4 SV=1
M7TP10	Putative glycosyl hydrolase protein	2	*Botrytis cinerea*	tr|M7TP10|M7TP10_BOTF1 Putative glycosyl hydrolase protein OS=*Botryotinia fuckeliana* (strain BcDW1) OX=1290391 GN=BcDW1_6097 PE=4 SV=1
M7UWR2	Putative glycoside hydrolase family 12 protein	2	*Botrytis cinerea*	tr|M7UWR2|M7UWR2_BOTF1 Putative glycoside hydrolase family 12 protein OS=*Botryotinia fuckeliana* (strain BcDW1) OX=1290391 GN=BcDW1_3087 PE=3 SV=1
Q6IVV6	Endo-beta-1,4-glucanase	2	*Botrytis cinerea*	tr|Q6IVV6|Q6IVV6_BOTFU Endo-beta-1,4-glucanase OS=*Botryotinia fuckeliana* OX=40559 GN=cel5A PE=3 SV=1
M7TIL4	Putative serine carboxypeptidase s28 protein	2	*Botrytis cinerea*	tr|M7TIL4|M7TIL4_BOTF1 Putative serine carboxypeptidase s28 protein OS=*Botryotinia fuckeliana* (strain BcDW1) OX=1290391 GN=BcDW1_8056 PE=4 SV=1
M7TU03	Putative glutaminase protein	2	*Botrytis cinerea*	tr|M7TU03|M7TU03_BOTF1 Putative glutaminase protein OS=*Botryotinia fuckeliana* (strain BcDW1) OX=1290391 GN=BcDW1_6591 PE=4 SV=1
M7TUB5	Putative gmc oxidoreductase protein	2	*Botrytis cinerea*	tr|M7TUB5|M7TUB5_BOTF1 Putative gmc oxidoreductase protein OS=*Botryotinia fuckeliana* (strain BcDW1) OX=1290391 GN=BcDW1_4158 PE=4 SV=1
M7TXJ0	Putative-binding protein	2	*Botrytis cinerea*	tr|M7TXJ0|M7TXJ0_BOTF1 Putative-binding protein OS=*Botryotinia fuckeliana* (strain BcDW1) OX=1290391 GN=BcDW1_5446 PE=4 SV=1
M7UBP5	Putative carbohydrate esterase family 8 protein	2	*Botrytis cinerea*	tr|M7UBP5|M7UBP5_BOTF1 Putative carbohydrate esterase family 8 protein OS=*Botryotinia fuckeliana* (strain BcDW1) OX=1290391 GN=BcDW1_198 PE=4 SV=1
M7UIF6	Putative fad binding domain-containing protein	2	*Botrytis cinerea*	tr|M7UIF6|M7UIF6_BOTF1 Putative fad binding domain-containing protein OS=*Botryotinia fuckeliana* (strain BcDW1) OX=1290391 GN=BcDW1_8122 PE=4 SV=1
M7UUR9	Putative 3-phytase a protein	2	*Botrytis cinerea*	tr|M7UUR9|M7UUR9_BOTF1 Putative 3-phytase a protein OS=*Botryotinia fuckeliana* (strain BcDW1) OX=1290391 GN=BcDW1_3765 PE=3 SV=1
M7U0T8	Uncharacterized protein	2	*Botrytis cinerea*	tr|M7U0T8|M7U0T8_BOTF1 Uncharacterized protein OS=*Botryotinia fuckeliana* (strain BcDW1) OX=1290391 GN=BcDW1_4127 PE=4 SV=1
